# First report of kerion (tinea capitis) caused by combined *Trichophyton mentagrophytes* and *Microsporum canis*

**DOI:** 10.1016/j.mmcr.2020.05.002

**Published:** 2020-05-19

**Authors:** Xinyu Yang, Xiuyan Shi, Wei Chen, Yabin Zhou, Michail S. Lionakis, Dimitrios P. Kontoyiannis, Wei Liu

**Affiliations:** a Department of Dermatology and Venereology, Peking University First Hospital, National Clinical Research Center for Skin and Immune Diseases, Research Center for Medical Mycology, Peking University, Beijing Key Laboratory of Molecular Diagnosis on Dermatoses, Beijing, 100034, China; b Fungal Pathogenesis Section, Laboratory of Clinical Immunology and Microbiology, National Institute of Allergy and Infectious Diseases, National Institutes of Health, Bethesda, MD, 20892, USA; c Department of Infectious Diseases, Infection Control and Employee Health, The University of Texas MD Anderson Cancer Center, Houston, TX, 77030, USA

**Keywords:** Kerion, Mixed infection, Mycological examinations, Dermoscopy, *Trichophyton mentagrophytes*, *Microsporum canis*

## Abstract

A 5-year-old boy was presented with large ulcer accompanied by surrounding follicular pustules on the left parietal scalp. Dermoscopy showed “comma” and dystrophic broken hairs. Fungal culture showed mixed growth of two types of colonies. *Trichophyton mentagrophytes* and *Microsporum canis* were identified by using mycological examinations. To our knowledge, this is the first case of kerion caused by the combined *Trichophyton mentagrophytes* and *Microsporum canis*. Treatment with oral terbinafine for 2 months was effective.

## 1 Introduction

Tinea capitis (TC) is a widespread scalp infection caused by dermatophytes, occurring predominantly in children. The distribution of causative species of dermatophytes varies greatly in different geographic regions. For example, *Microsporum canis* remains the most common organism in Europe, South America and China [1,2], while *Trichophyton tonsurans* is the most prevalent agent in the UK and North America [1]. Kerion is an inflammatory type of TC and occurs commonly in rural areas with poor hygienic conditions and prepubertal children are more easily affected. Recently, the incidence of kerion is increasing in urban areas and pets are likely to be the most important sources of infection [3]. And adult patients with immunosuppression due to leukemia, organ transplant and the use of immunosuppressants may be also prone to developing kerion [3]. A single species of dermatophytes, including *M. canis*, *Trichophyton violaceum* or *T. tonsurans*, has been isolated more frequently from kerion in recent years [4,5]. It has been observed that kerion in Europe is increasingly associated with *T. tonsurans* [5], while those in southeastern and northwestern of China are more related with *T. violaceum* [2]. In this report, we describe the first case of kerion caused by combined *Trichophyton mentagrophytes* and *M. canis.*

## 2 Case

**Fig. 1:**
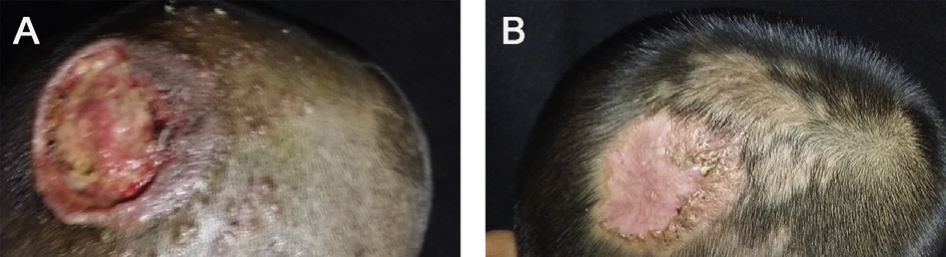
Clinical presentation. (A) Before treatment. Shown is a large 3 × 4 cm ulcer accompanied by several peripheral follicular pustules on the left parietal scalp. (B) After treatment. The lesion improved significantly after 2 months of treatment, leaving residual scarring alopecia.

A 5-year-old boy presented with a large ulcer accompanied by surrounding follicular pustules on the left parietal scalp (day 0). Two weeks prior, the lesions appeared initially as pruritic follicular pustules, which increased gradually in size and formed an abscess. Before appearance of these lesions, he had experienced scalp scratch. He initially underwent incision and drainage of the abscess in other hospital and was treated with oral amoxicillin. A large ulcer appeared subsequently at the primary pustule site, and he presented to our hospital for further treatment. Besides, he lives in the rural area and keeps pets including cat and rabbit. Physical examination revealed a 3 × 4 cm ulcer with several peripheral follicular pustules on the left parietal scalp (Fig. 1A). Left cervical lymphadenopathy was present. The hairs around the ulcer were plucked easily. Findings from complete blood cell count, liver function and renal function were unremarkable. The result of bacterial culture of the secretion was negative (day +2).

**Fig. 2:**
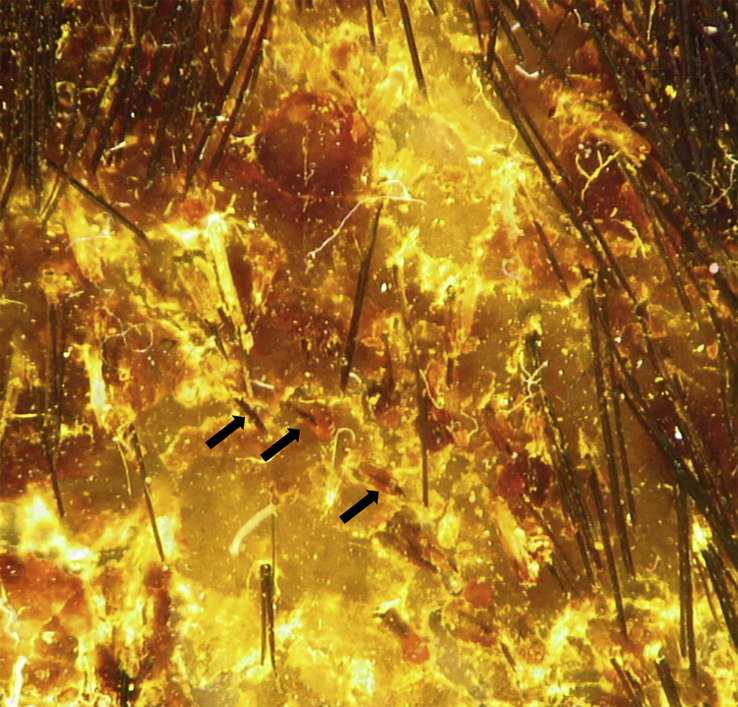
Dermoscopic image of scalp lesions. Large patches of erythema, scales, “comma” hairs (arrowhead), and dystrophic broken hair with white hair casts.

**Fig. 3:**
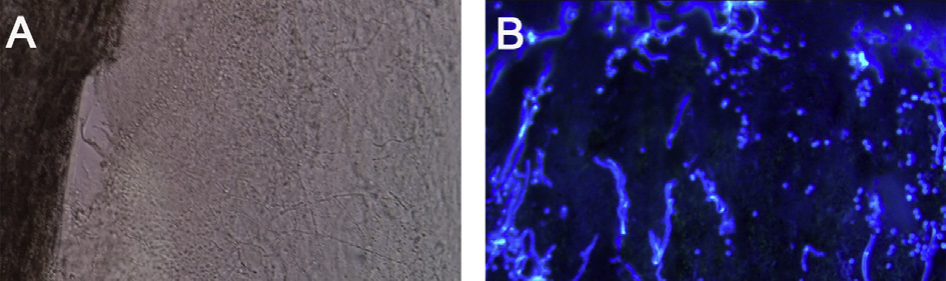
Microscopic image of scalp lesions. Numerous ectothrix spores and hyphae without (A) and with calcofluor white staining (B), respectively (original magnification × 100).

**Fig. 4:**
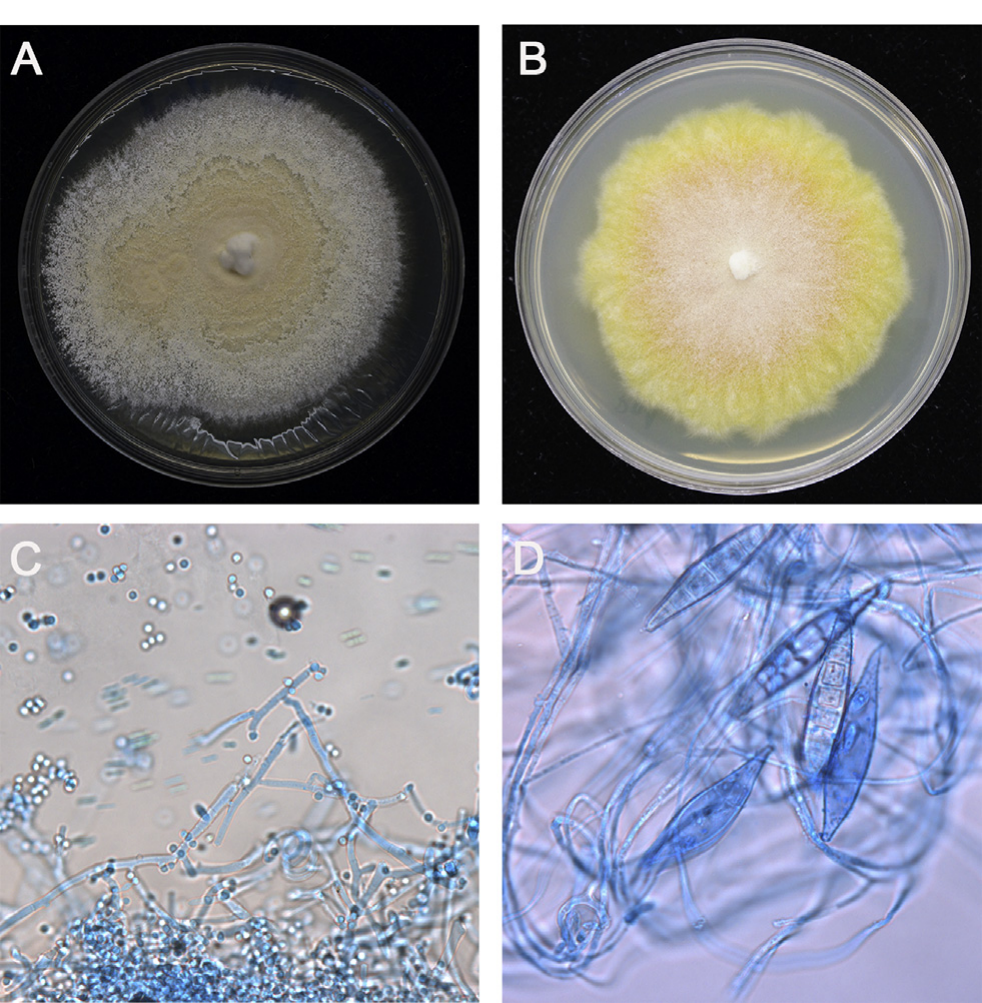
Macroscopic and microscopic characteristics of two isolates. (A) White cotton-like and yellowish (B) colonies cultured on Sabouraud dextrose agar, respectively (grown for 14 days at 28°C). The white cotton-like colony showed grape-like clusters, spherical microconidia alongside the hyphae (C), while the yellowish colony showed spindle-shaped macroconidia with thick walls and thinner septa alongside undifferentiated hyphae (D) with lactophenol cotton blue staining (original magnification × 400). (For interpretation of the references to colour in this figure legend, the reader is referred to the Web version of this article.)

Dermoscopic examination showed large patches of erythema, scales, “comma” hairs, and dystrophic broken hairs with white hair casts near the scalp by using a hand-held dermoscope connected with digital camera (day +1) (Fig. 2). Direct microscopic examination of the scrapings from the lesions revealed numerous ectothrix spores and hyphae by using 10% potassium hydroxide (KOH) with calcofluor white staining (day +1) (Fig. 3A and B). Fungal culture of the ulcer drainage showed mixed growth of two types of colonies (day +7), which were further subcultured on Sabouraoud dextrose agar for 14 days. One of the colonies had a white cotton-like appearance and the other was yellowish (Fig. 4A and B). Under the microscopy, the isolate with white cotton-like appearance showed grape-like clusters, spherical microconidia alongside the hyphae, while the yellowish spindle-shaped macroconidia with thick walls and thinner septa alongside undifferentiated hyphae by using lactophenol cotton blue staining (day +21) (Fig. 4C and D). Genomic DNA was extracted using the Biospin Fungus Genomic DNA Extraction Kit (Bioer Technology Ltd, Hubei, China) according to the manufactures' instructions. Molecular identification was performed by sequencing of the internal transcriber spacer (ITS) regions, which were amplified by polymerase chain reaction (PCR) using the primers ITS1 (5′-TCCGTAGGTGAACCTGCGG-3′) and ITS4 (5′-TCCTCCGCTTATTGATATGC-3′) in a reaction volume of 25 μl containing 20 ng of genomic DNA, 0.08 μM each of the primers, 12.5 μl 2 × Taq PCR MasterMix (Tiangen Biotech Ltd; Beijing, China). The reaction cycles included an initial denaturation step at 95°C for 5 min; 35 cycles of 95°C for 30 s, 60°C for 30 s, and 72°C for 1 min; followed by a final extension at 72°C for 5 min. Amplification product was visualized using gel electrophoresis and sequenced by BGI Company (Beijing, China). Alignment of ITS sequences were performed on the CBS database (http://www.wi.knaw.nl/Collections/BioloMICSSequences.aspx?expandparm=f&file=FUNGI&file=FUNCBS&file=YEASTseq&file=MOLDseq&file=UNITE&file=Yeast&file=Fusarium&file=Dermato&file=indoor&file=Morch&file=ITSSeq&file=Bipolaris&file=CGattii&file=CNeof&file=PJ&file=Scedo&file=FunBOLD&file=PYCC&file=EFSeq&file=Mycokey&file=PasteurMOULD&file=PasteurYEAST). Molecular identification for these 2 isolates suggested identity to *T. mentagrophytes* and *M. canis*. Taking these data together, the two isolates were identified as *T. mentagrophytes* and *M. canis*, respectively. Therefore, a diagnosis of kerion caused by a combination of *T. mentagrophytes* and *M. canis* was finally established.

The patient was treated with oral terbinafine (125 mg per day) combined with topical 2% ketoconazole cream for two months. In parallel, oral prednisone (20 mg per day) was administered for two weeks to suppress inflammation. The lesions improved significantly, leaving residual scarring alopecia (Fig. 1B), and no relapse was noted at a 4-month follow-up visit.

## 3 Discussion

TC is a common scalp disease in the worldwide, most often caused by *T. tonsurans* or *M. canis* [5]. Kerion represents the inflammatory variant of TC caused by the hypersensitivity reaction to the causative dermatophyte and is primarily affecting children between five-ten years of age [[6], [7], [8]]. Kerion is characterized by suppurative and painful plaque associated with purulent drainage and regional lymphadenopathy. Kerion is often misdiagnosed, leading to delay proper treatment and permanent scarring alopecia [9]. Therefore, early diagnosis and timely antifungal treatment are very important.

Dermoscopic examination is a rapid, non-invasive and painless method for the diagnosis of TC [[10], [11], [12]]. In addition, dermoscopy is particularly important in differential diagnosis between TC and alopecia areata [11]. “Comma” and “corkscrew” hairs are the classic findings of TC under dermoscopy [13]. Comma-shaped hairs are characterized by a sharp slanting end, homogeneous thickness, and pigmentation of hair shaft, which represent an intermediate stage before formation of dystrophic hairs. And “corkscrew” hairs show more exaggerated coiled appearance of the hair shaft [14]. Mycological examinations are considered to be the gold standard for diagnostic method of TC [3]. And the samples of hairs, scalp and hair scales are examined directly by microscope as well as cultured on Sabouraud dextrose agar. However, fungal culture is time-consuming and usually requires two weeks for results [9].

This patient was initially misdiagnosed as bacterial infection and underwent incision and drainage of abscess that made TC aggravated. After mycological examinations in our hospital, kerion caused by combined *T. mentagrophytes* and *M. canis* was diagnosed.

The aim of the treatment is to eradicate the organism to achieve both clinical and mycological cure as quickly and safely as possible [5]. Treatment for TC relies on the use of systemic antifungal agents since topical agents cannot penetrate the hair shaft [1]. Griseofulvin is the first-line drug and it is particularly useful for *Microsporum* infections [5,15]. However, it is not available in pediatric form in many countries and adverse effects limit its use at present [1,5]. Terbinafine shows antifungal activity against all dermatophytes, but has much higher efficacy against *Trichophyton* spp. than *Microsporum* spp [5,15]. Terbinafine is considered to be the optimal choice and is often used as first-line for TC in children [1,5]. Itraconazole has activity against both *Microsporum* and *Trichophyton* species, and is commonly used as second-line therapy [5]. Hepatotoxicity is the main adverse effect of the systemic antifungal agents. Therefore, monitoring liver function is necessary during treatment. It is suggested that the use of topical agents can be an adjuvant therapy to decrease treatment duration and reduce transmission of spores [15,16]. In addition, oral corticosteroids for kerion may reduce inflammation response and the risk of permanent scarring or alopecia [16]. Therefore, we adopted the treatment of oral terbinafine combined with 2% ketoconazole cream, and a short course of corticosteroids, resulting in clinical and mycological cure.

Recently, the occurrence of mixed infection is a growing public health problem, including bacteria and fungi such as *Mycobacterium leprae* and *Cryptococcus* [17], different species of fungi such as *Aspergillus fumigatus* and *Candida albicans* [18]. TC is rarely caused by several species of dermatophytes, which has also been described previously [19,20]. Our patient represents, to our knowledge, the first description of kerion caused by co-infection with by *T. mentagrophytes* and *M. canis*. Therefore, we conclude that clinicians should be aware of TC that can be rarely caused by mixed dermatophyte infection, and that dermoscopic and mycological examinations are clinically useful in differentiating between kerion and other scalp diseases.

## Declaration of competing interest

There are none.
